# Plant Genotype to Phenotype Prediction Using Machine Learning

**DOI:** 10.3389/fgene.2022.822173

**Published:** 2022-05-18

**Authors:** Monica F. Danilevicz, Mitchell Gill, Robyn Anderson, Jacqueline Batley, Mohammed Bennamoun, Philipp E. Bayer, David Edwards

**Affiliations:** ^1^ School of Biological Sciences and Institute of Agriculture, University of Western Australia, Perth, WA, Australia; ^2^ School of Physics, Mathematics and Computing, University of Western Australia, Perth, WA, Australia

**Keywords:** machine learning, plant phenotyping, phenotype prediction, plant breeding, big data

## Abstract

Genomic prediction tools support crop breeding based on statistical methods, such as the genomic best linear unbiased prediction (GBLUP). However, these tools are not designed to capture non-linear relationships within multi-dimensional datasets, or deal with high dimension datasets such as imagery collected by unmanned aerial vehicles. Machine learning (ML) algorithms have the potential to surpass the prediction accuracy of current tools used for genotype to phenotype prediction, due to their capacity to autonomously extract data features and represent their relationships at multiple levels of abstraction. This review addresses the challenges of applying statistical and machine learning methods for predicting phenotypic traits based on genetic markers, environment data, and imagery for crop breeding. We present the advantages and disadvantages of explainable model structures, discuss the potential of machine learning models for genotype to phenotype prediction in crop breeding, and the challenges, including the scarcity of high-quality datasets, inconsistent metadata annotation and the requirements of ML models.

## Introduction

The expansion of genome sequencing technology has led to a rapid growth in plant genomic resources, including advanced genome and pangenome assemblies, providing a better understanding of plant genetic variation. Genotyping methods including genome resequencing (Morrell et al., 2011; Hu et al., 2018; Juliana et al., 2018) and single nucleotide polymorphism (SNP) arrays (Wang et al., 2014; Mason et al., 2017) have supported the expansion of crop genomic resources, assisting the identification of genetic variations related to agronomic traits. The emergence of plant pangenomes as a reference better represents the genomic variability within a species (Bayer et al., 2020; Danilevicz et al., 2020; Golicz et al., 2020), allowing researchers to expand genotypes from SNPs and indels to include gene presence absence variation, a structural variation that has been associated with crop disease resistance and stress tolerance (Golicz et al., 2016; Montenegro et al., 2017). Plant breeders now have a wide variety of tools available to assess genetic variation, however the most efficient way to apply these for crop improvement is often unclear (Heffner et al., 2009; Scheben et al., 2017; Nuccio et al., 2018). Genomic selection (GS) using best linear unbiased prediction (BLUP) was developed for livestock breeding (Meuwissen et al., 2001), but has seen success in plant breeding (Cooper et al., 2014; Gaffney et al., 2015; Crossa et al., 2017; Lebedev et al., 2020; Ukrainetz and Mansfield, 2020). This approach takes a subpopulation of breeding material, and through linear modelling, estimates the contribution of each SNP to phenotypes of interest. Due to the simplicity of the modelling, BLUP is straightforward to implement, and the contribution of each SNP to the phenotype is relatively easy to calculate. However, compared to animal breeding, genomic selection in plants has to account for the greater genotype by environment interactions, and requires the addition of appropriate multi-environment trial data (Voss-Fels et al., 2019).

Machine Learning (ML) and Deep Learning (DL) algorithms are more complex than linear prediction models and can discover non-linear relationships within datasets. The use of complex ML and DL models to associate plant genotypes with phenotypes is gaining popularity, with an increasing number of publications predicting a wide variety of agronomic traits, including yield, days to heading and thousand kernel weight (Montesinos-López A. et al., 2018, Montesinos-López et al., 2018 O. A.; Ma et al., 2018; Crossa et al., 2019; Khaki et al., 2019; Grinberg et al., 2020). Random forests, support vector machines and artificial neural networks may more easily capture the complex relationships between genotype, phenotype and environment compared with previous methods due to their non-linearity, and ML and DL have significant potential to support plant breeding. Here we review the progress that has been made applying ML and DL methods, and how to interpret their results within a biological context.

## Machine Learning Methods for Genotype to Phenotype Prediction

Genotype to phenotype prediction has expanded with the application of GS. Genomic Best Linear Unbiased Prediction (GBLUP), a linear based modelling system has been used extensively in GS (Meuwissen et al., 2001), as have Bayesian systems (Pérez et al., 2010). Despite the successes of linear methods in GS, they can run into challenges due to the high dimensionality of marker data versus the number of individuals, and the presence of complex relationships that are difficult to account for (Crossa et al., 2017).

**FIGURE 1:**
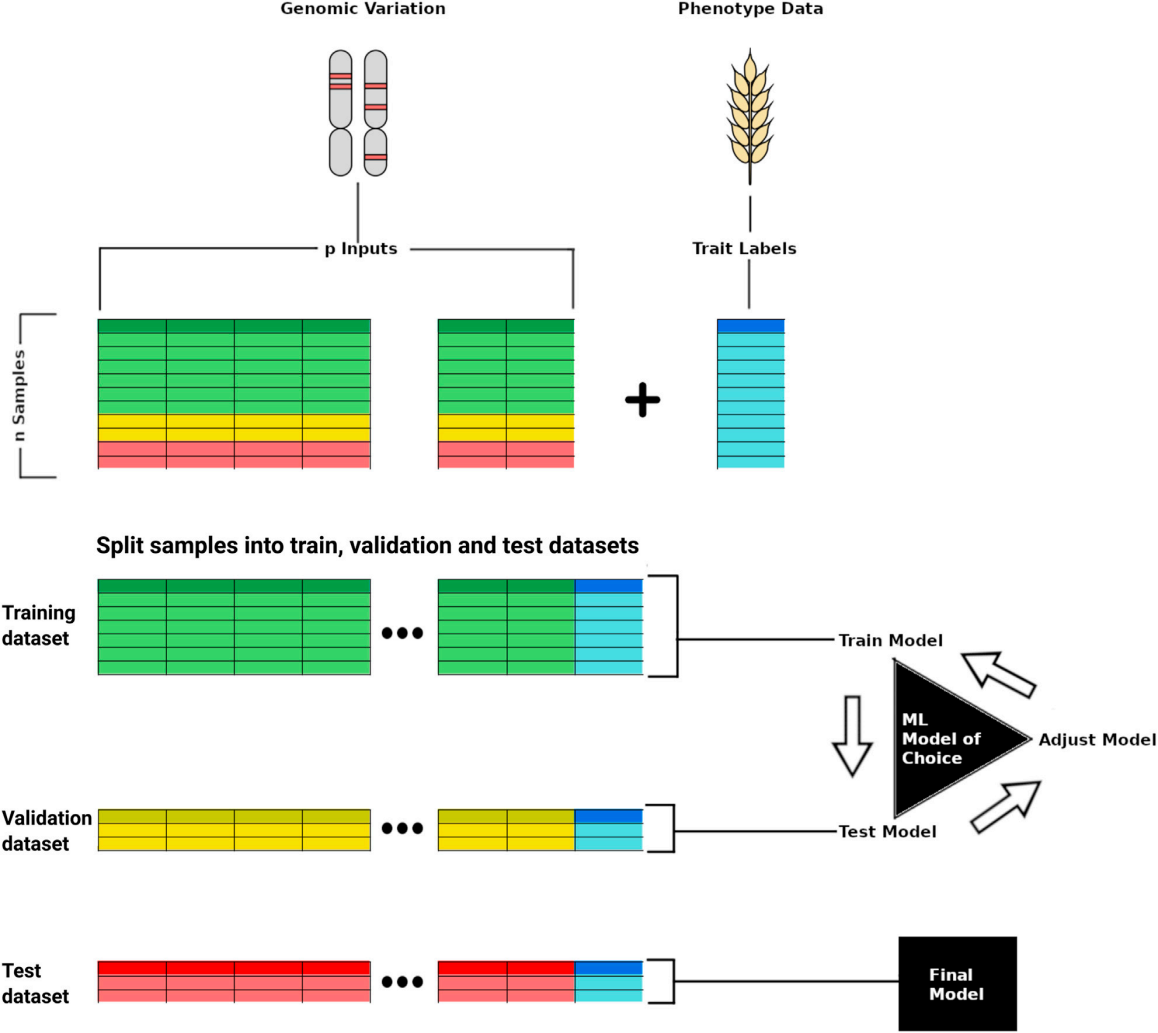
Scheme representing training a deep learning model for phenotype prediction based on genomic variation data. In the top, the genomic variation data of p samples is used as input whereas the phenotypic trait (e.g., yield) is used as a label for training the model. The genomic and phenotypic data is split into training, validation, and test datasets, with the first two employed for model development, and the last used last for testing the trained model capacity to predict a target trait from unseen input data.

To improve on linear models in GS, there has been an increased use of nonlinear methods such as ML and DLmodels to predict plant phenotypes (Montesinos-López et al., 2021a). Nonlinear methods have been theorised to be better able to capture small interactions between markers, account for environment interactions and generate predictions with higher accuracy for data with high dimensionality (Pérez-Rodríguez et al., 2012; Cuevas et al., 2016). The ML and DL architectures can also include multimodal data and data types that are not suited to simple tabular formats (Figure 1). For example, the ML method random forest can capture patterns in high dimensional data to deliver accurate predictions and can also take into account non-additive effects (Heslot et al., 2012). Its use as a model for genomic selection has also demonstrated superior performance in comparison to linear models such as Bayesian Least Absolute Shrinkage and Selection Operator (LASSO) (Ornella et al., 2014) and Ridge Regression BLUP (RR-BLUP), depending on the genetic architecture of the predicted trait (Spindel et al., 2015). Other ML models that have shown potential for genomic selection include convolutional neural networks and feed forward deep neural networks that can outperform linear methods with correct optimisation of hyperparameters (Ma et al., 2018; Sandhu et al., 2020). Multi-trait DL models can help understand the relationship between related traits for improved prediction (Montesinos-López O. A. et al., 2018), and ensemble models that are a powerful way of combining multiple ML methods that may produce weaker predictions by themselves (Jubair and Domaratzki, 2019; Banerjee et al., 2020).

Whether ML and DL approaches improve prediction compared to traditional GBLUP for genomic selection is still unclear. Several publications compare DL models with linear ML models, but not with GBLUP directly, and many breeding methods use BLUP values, whereas ML and DL studies tend to predict single traits. In an example where GBLUP was compared to DL methods for multi-trait prediction, when the genotype by environment interaction term was included in the GBLUP modelling, it outperformed DL across 8 of the 9 datasets, whereas when it was not included, the DL model outperformed GBLUP in 6 of the 9 datasets (Montesinos-López A. et al., 2018). Another study observed that their DL model outperformed linear and Bayesian prediction models when predicting under strong epistatic interactions, using SNPs from octoploid strawberry and tetraploid blueberry (Zingaretti et al., 2020). As with many problems that involve algorithm choice, the no free lunch theorem suggests that no one algorithm will perform the best across all problems, and this seems to be the case for genomic prediction (Azodi et al., 2019). DL is an efficient method to extract representative features from large datasets, with the capacity to account for feature interaction effects. However, conventional ML methods and mixed linear models are still well suited to deal with limited datasets, in many cases generating more accurate predictions than DL models. We propose that phenotype prediction should be expanded beyond GBLUP methods to ensure sufficient models are evaluated for each genomic selection problem.

## Genetic Variation Representation

The most common form of encoding whole genome SNP data for ML and DL, is to use one hot encoding, where each SNP position is represented by four columns, each one representing the four bases of DNA: A, T, C, and G. Presence of the base at each position is indicated by a 1 in this column, whereas absence is indicated by 0 (Zou et al., 2019). In this way a letter can be encoded as a binary representation, suitable for ML and DL processes, that only accept numeric input. SNP one hot encoding is one of the most common data representations for DNA sequence data in phenotype prediction (Montesinos-López A. et al., 2018, Montesinos-López et al., 2018 O. A.; Ma et al., 2018; Khaki et al., 2019; Grinberg et al., 2020).

In ML and DL, the number of features is usually lower than the number of samples, however it is common in trait association studies that the number of features significantly outnumbers the samples. Genotyping usually generates large numbers of genetic markers and phenotyping large cohorts of plants can be prohibitively expensive, restricting the number of samples. Feature selection, minor allele frequency and genome wide association study (GWAS) can be applied to reduce the dimensionality of datasets and remove excess information, presenting varying degrees of popularity in plant breeding. In human research, some studies have used minor allele frequency, position within promoter regions (Yin et al., 2019), selective SNP number reduction, and integrated transcription data (Zhou et al., 2019), where previous association results are used to strategically reduce SNP numbers. Other strategies to reduce SNP numbers include a focus on rare variants, where loss of function variants have been noted for their influence in phenotype (Monroe et al., 2020) and using GWAS to select regions of interest, though this has had mixed results (Spindel et al., 2016; Rice and Lipka, 2019; Jeong et al., 2020).

Genotype encoding in plants has been mostly limited to SNP encoding, however there are other forms of genomic variation, and different ways of encoding genetic variation data that could be employed for phenotype prediction. A recent article used methylation at cytosine positions to predict traits in *Populus balsamifera*, input cytosines were encoded using variable importance scores and individual methylation intensity, The results show that biomass and physiochemical wood traits can be modelled using methylation data and DL (Champigny et al., 2020). Outside of plant research, genomic k-mers were used to encode genomic variability and employed to predict antibiotic resistance in multiple species of pathogenic bacteria, using classification and regression trees and set covering machines algorithms for interpretability (Pérez et al., 2010; Drouin et al., 2019). In Chen et al. (2019), rare genetic variants from a *Mycobacterium tuberculosis* dataset were grouped by locus, using binary encoding to account for mutation presence/absence and feature labelling to encode the type of mutation (e.g., SNP, insertion/deletion, change in frameshift within a coding region). The dataset was used to predict antibiotic resistance in the pathogen. improving prediction for both logistic regression models and neural networks. The expansion of plant pangenome assemblies provides an opportunity to include gene presence/absence variation in trait prediction, encoded in a similar way to SNPs (Golicz et al., 2016; Jin et al., 2016; Hurgobin and Edwards, 2017; Gao et al., 2019). The way researchers choose to encode the genomic information and use the associated data (environmental or gene expression data) is an important aspect of trait association analysis, and a wider variety of encoding methods should be explored.

## Integrating High Throughput Phenotyping Into Genotype to Phenotype Models

Genotype to phenotype models are commonly applied to sparsely collected phenotypic traits such as plant height and germination rate, and these traits are often hand-collected, potentially introducing bias and increasing experimental cost. In contrast, high throughput phenotyping allows monitoring hundreds or thousands of plants under field and greenhouse conditions (Tattaris et al., 2016; Shakoor et al., 2017), performing non-destructive measurement of traits such as biomass (Quirós Vargas et al., 2019; Marino and Alvino, 2020), plant height (Anderson et al., 2020), wheat spike count (Hasan et al., 2018; Xiong et al., 2019), and disease (De Castro et al., 2015; Nagasubramanian et al., 2018). The increased density of phenotypic data produced by high throughput phenotyping enables researchers to dynamically measure changes in plant growth, evaluating the impact of genomic variation at different developmental stages. For example, a study using images collected weekly by an unmanned aerial vehicle observed that two SNPs related to maize height had a larger effect during early growth (10–25 cm), with their contribution to this phenotype decreasing towards the end of the season (Adak et al., 2021). In rice, high throughput imagery was used to calculate six vegetation indexes that were used as input traits for the identification of eight quantitative trait loci (QTLs), one of which was corroborated by another analysis using ground-truth agronomic data (Naito et al., 2017). Similarly, vegetation indices and canopy temperature were employed in the identification of QTLs related to abscisic acid concentration and other physiological traits in cotton using inclusive composite interval mapping for multi-environment trials (Pauli et al., 2016). Vegetation indices and temperature were also used to support genomic selection for grain yield in wheat (Rutkoski et al., 2016). These studies show the potential of incorporating raw or extracted features from image data to detect genomic variation associated with desirable traits. Intermediate phenotypes such as transcriptome, proteome or metabolome data can also be associated in a multidimensional dataset, providing a more detailed description of the plant response to environmental conditions, and potentially increasing phenotype prediction accuracy (de Abreu E Lima et al., 2018; Voss-Fels et al., 2019; Scossa et al., 2021).

**FIGURE 2:**
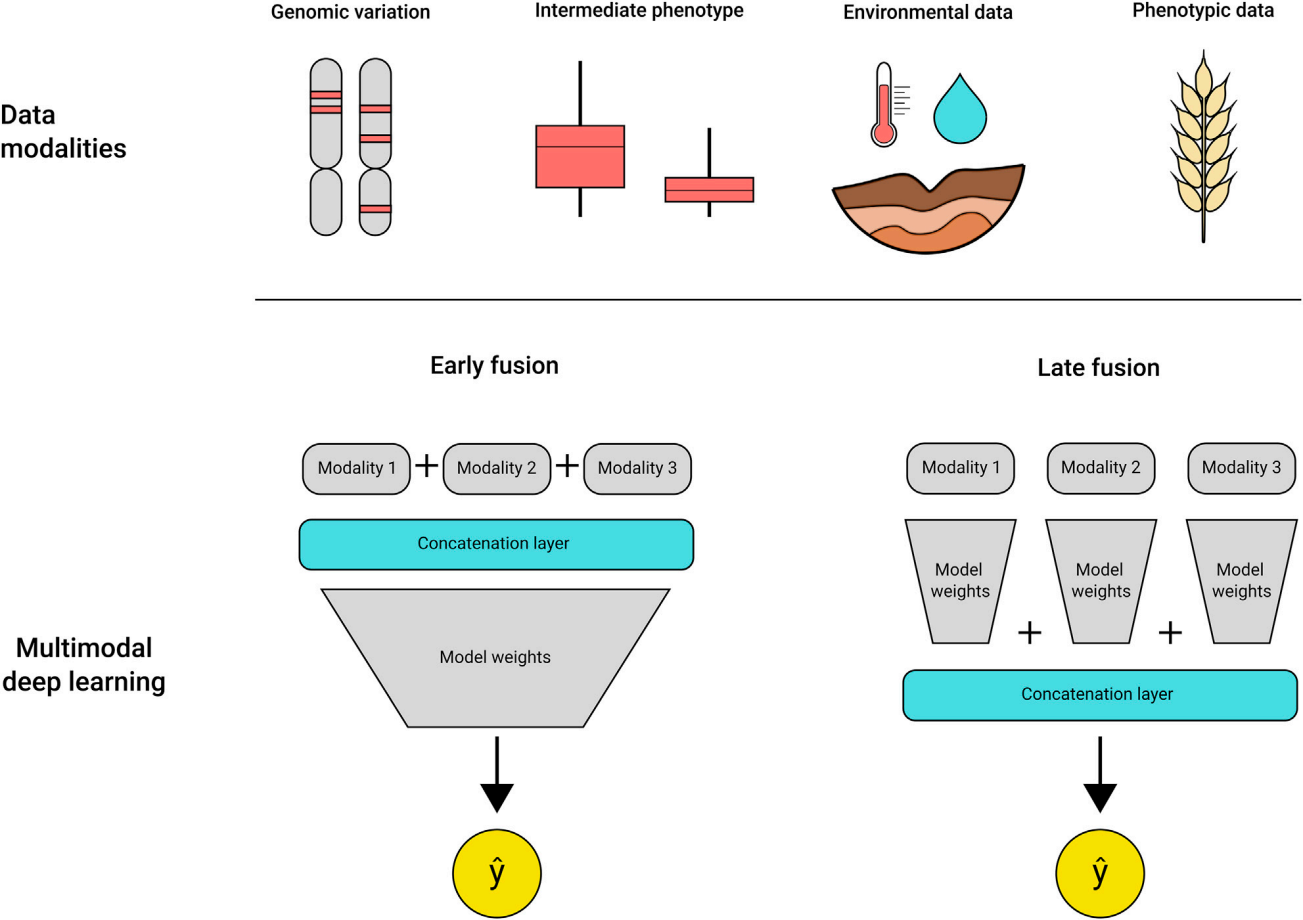
Multimodal DL models can use a variety of inputs as training data, as shown in “data modalities” at the top. The different data types can be fused early in the training process by using a concatenation layer that integrates the multiple data types in a single file per sample, called early fusion. Alternatively, the concatenation step may be deployed at the final layers of the model, merging the weights from each specialised module before outputting a prediction.

The addition of multidimensional datasets can increase the complexity of analysis exponentially, requiring algorithms that are able to uncover the relationships between the data types and the target trait. DL models have shown success in dealing with complex multimodal datasets, being effectively applied to generate image descriptions (Hossain et al., 2021), predict injuries in sports (Song et al., 2021) and disease detection from medical datasets (Khan et al., 2020; McKenzie et al., 2020; Fan et al., 2021). Recently, several studies were published using DL for trait prediction using high throughput plant phenotyping images as input. These take advantage of convolutional neural networks for extracting the spectral features of the leaf reflectance for disease classification (Picon et al., 2018; Nagasubramanian et al., 2019), wheat spike segmentation and counting (Misra et al., 2020), and QTLs related to root architecture traits (Pound et al., 2017). A wide diversity of DL architectures that have been developed to address problems in other areas of research can be adapted for use in crop breeding. For example, multimodal DL models are composed of multiple models, each trained using a single input type (e.g., rainfall, soil measurements, genetic data, hyperspectral imagery), or a single model trained on concatenated multimodal data (Figure 2). The different modalities contribute to enrich the available features for model learning, contributing to an improved final prediction (Baltrusaitis et al., 2019; Khaki et al., 2019; Gadiraju et al., 2020; Hoang Trong et al., 2020; Maimaitijiang et al., 2020). The use of multimodal models and other DL architectures, such as recurrent neural networks and graph neural networks, are still largely unexplored in genotype to phenotype predictions, but present a powerful alternative to traditional statistical methods.

**FIGURE 3:**
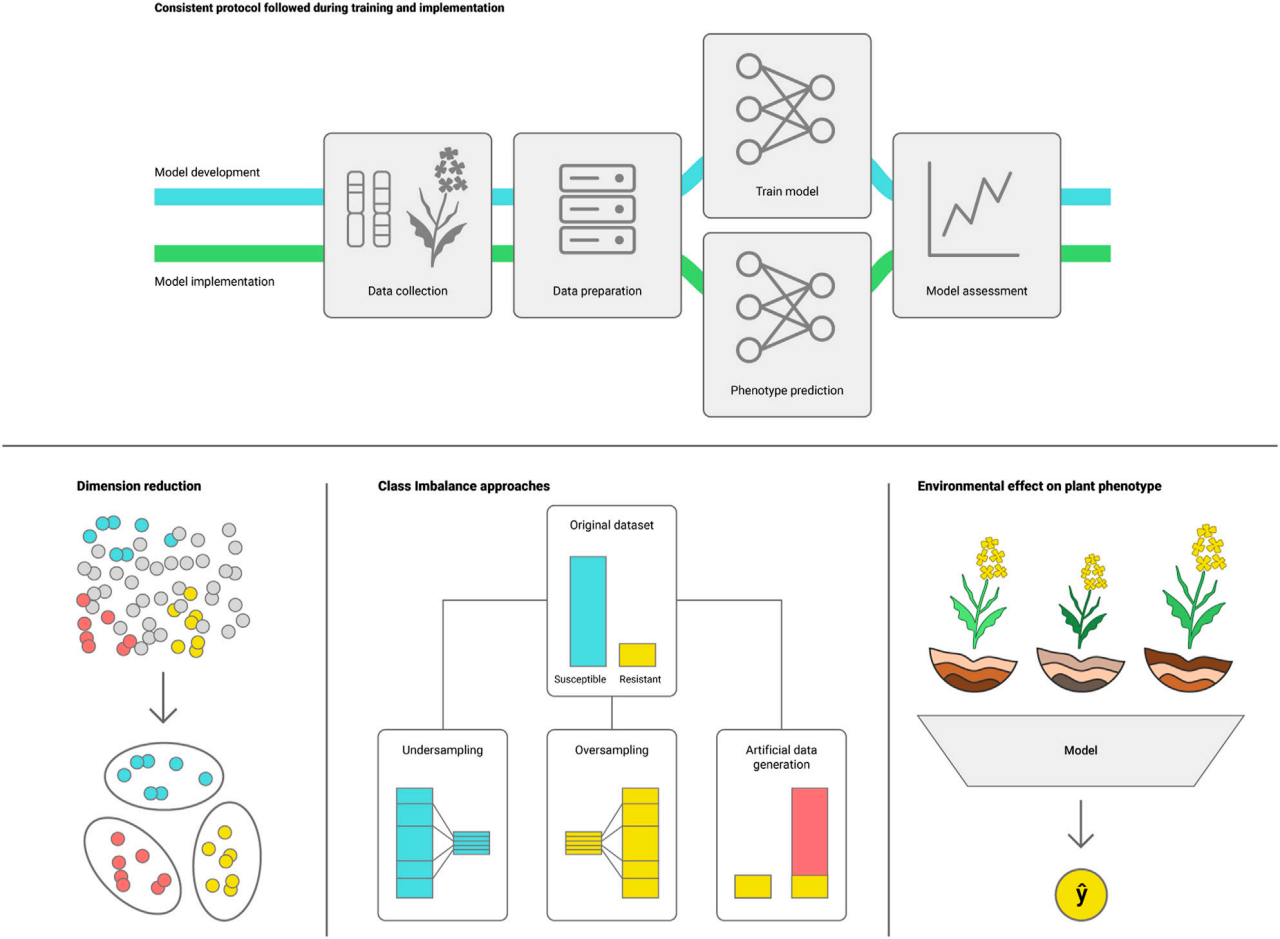
Potential challenges for deploying DL models for plant phenotype prediction: I) consistent protocols for development and implementation, ii) reduce dataset dimensionality, which may conceal valuable information, iii) reduce class representation imbalance and iv) account for environmental variations between conditions for plant growth in the training and deployment datasets.

Challenges in deploying DL models emerge mainly from plant phenotypic plasticity, as plants present a wide range of phenotypes depending on the environmental conditions (Nicotra et al., 2010). The effectiveness of ML/DL models also depends on appropriately tuning the model hyperparameters to the target task, with many packages designed to assist tuning such as Optuna and HyperOpt (Bergstra et al., 2013; Akiba et al., 2019). Nonetheless, tuning hyperparameters for DL models tends to be more computationally intensive than conventional ML. Below we list some challenges that arise from phenotype variability that may need to be addressed to effectively use ML for genotype to phenotype prediction (Figure 3).

i) Consistent protocol for data collection and processing during training and model deployment (Hagiwara et al., 2020; Mårtensson et al., 2020). Because DL models learn directly from the dataset, varying the methods for data collection and processing can add noise causing the model to underperform. It is important to maintain a consistent protocol for data handling, as well as regular assessment to ensure that the model remains suitable for the task;
ii) Avoid the curse of dimensionality (Altman and Krzywinski, 2018). High throughput phenotyping platforms, hyperspectral cameras and pangenome assemblies can generate massive amounts of data, making it harder for the model to define which data points are representative of the trait. Feature selection algorithms can assist in selecting the most representative data subset for training the DLmodel (Cen et al., 2016; Khaki and Wang, 2019);
iii) Data imbalance. Scarcity of samples representing a specific genotype or environment can introduce bias to the model. This can be addressed by employing sampling methods such as over and under sampling, or generative DL to build an artificially augmented dataset (Radford et al., 2015);
iv) Changes in environmental conditions (interannual weather variation, differences in agroecological zone and crop management practices) may impact model performance due to plant phenotypic plasticity. Environmental effects on the phenotype should be considered when defining the model validation and future applicability, and can be addressed by collecting data that mimic the conditions that the model will see when predicting the phenotype (Montesinos-López O. A. et al., 2018; Khaki et al., 2019; Shook et al., 2021).

As crop research advances, it is advisable that the models are updated with new data collected. ML and DL models are under intense research, with improvements and novel architectures being published regularly. Selecting the most appropriate architecture and addressing the challenges above will depend on the goal of the project and the data availability. Some data types benefit from complex DL models that draw complex non-linear relationships from the data, while others can achieve high performance using simpler tree-based models with greater potential for interpretability of the results. Nonetheless, the successful implementation of ML/DL workflows in crop breeding will require the familiarisation of breeders and other stakeholders to the models capabilities, so they can be used appropriately.

## Data-Driven Breeding Requires Structured Datasets

The prediction accuracy of machine learning models is intrinsically related to the quality of the dataset employed during development (LeCun et al., 2015). ML models calibrate their internal weights based on the data provided, requiring a representative dataset with accurate annotation (Sasidharan Nair and Vihinen, 2013; Schaafsma and Vihinen, 2018). Consequently, a frequent challenge for training robust ML models is the lack of appropriate datasets with enough data points and sample variability. It has been suggested that the scarcity of plant phenotype datasets is because these are either inefficiently shared with the community due to missing information and difficulties to find the public repository in which it is stored (Zamir, 2013; Lobet, 2017), or because the data is maintained in data silos with restricted access (Bayer and Edwards, 2020). A few international consortia such as the AgBioData (Harper et al., 2018) and Breeding API (Selby et al., 2019) are making an effort to share and transform breeding datasets to become more findable, accessible, interoperable and reusable (FAIR) (Wilkinson et al., 2016). However, a centralised platform for hosting and managing phenotypic datasets is needed to make data more widely available, similar to approaches used to share genomic data. Another aspect that prevents researchers from employing previously published datasets is the lack of standardised metadata descriptions, encompassing experimental design, data collection protocol, field management, environmental variables, and other information. The observed plant phenotype is the result of the conditions that the plant experiences, thus reusing previously published data requires that all the factors influencing the target trait are described for the user. The Minimum information about a plant phenotype project (MIAPPE) (Papoutsoglou et al., 2020) offers a resource to guide researchers on how to annotate metadata to increase the usability and interoperability of the datasets, but the development and application of suitable standards need to be expanded to support the growth in ML/DL applications.

**FIGURE 4:**
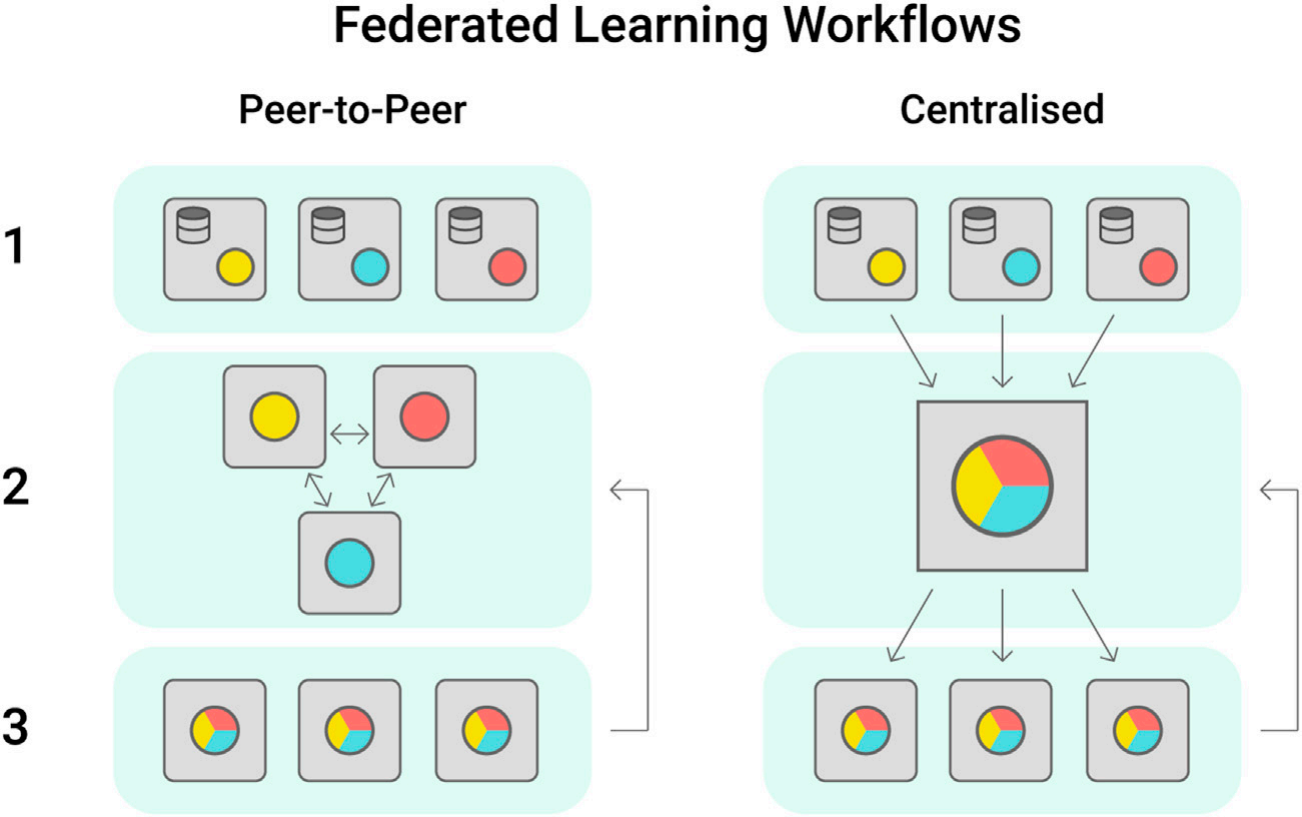
Federated learning workflows are most proposed in the peer-to-peer or centralised configurations. In Peer-to-peer, each institution trains a model locally using their own dataset, sharing the model trained weights with its partners. In this workflow, the models are aggregated by each institution as preferred. In the centralised scheme, the trained models are shared with one centralised cohort, which will aggregate the models received and share a singular version with the stakeholders.

A large number of high quality phenotypic datasets are generated each year during field trials led by industry, government or academic groups, however these often have controlled access to protect industry knowledge. An alternative approach to protect sensitive information while supporting collaboration towards data-driven breeding is the establishment of federated learning cohorts (Konečný et al., 2016). Within these, each participant institution trains the model with its own dataset and shares the updated model peer to peer or to a centralised server that will aggregate the models weights (Figure 4). The updated model parameters improve the baseline model, that is then shared among institutions (Yang et al., 2019). Federated learning has seen increasing application in digital health, where data sensitivity is a major issue (Brisimi et al., 2018; Lee et al., 2018; Rieke et al., 2020).

## Explainable (Interpretable) Machine Learning for Genotype to Phenotype Models

The increasing use of large scale data, such as genomic variation and high throughput imagery, have produced studies with highly accurate ML and non-ML prediction models (Montesinos-López et al., 2021b). However, building models capable of predicting a biological output can only be seen as one of the goals. Models should also attempt to address biological questions, which requires an understanding of how the models make predictions. The use of ML as a method to ask biological questions presents researchers with the problem of model explainability. Explainability in prediction models is a relatively new area for genomic prediction, as the main goal has often been to achieve optimum prediction performance, with model explainability less of a focus. This idea can be represented with GBLUP, one of the most widely implemented genomic selection methods. GBLUP offers researchers accurate prediction of breeding values but low explainability of its predictions, as estimating individual SNP effects can be difficult for genomic prediction datasets due to the “large-p little-n” problem (de Los Campos et al., 2013; Shen et al., 2013). As crop genomic prediction ability advances, it is becoming increasingly important for researchers to explain how a “black box” model has made predictions, to understand the biological implications, justify further research questions and support problem specific model improvement.

For genotype to phenotype prediction in crops, explainability provides the ability to identify important genomic markers and then apply these genomic markers to reduce the size of model inputs required to make further predictions. In Khaki and Wang (2019), analysis of feature importance determined that weather had a larger effect on crop yield than genotype, and models trained on a reduced selection of top ranking input features did not lead to a significant reduction in performance (Khaki and Wang, 2019). Soybean yield prediction models using genotype and other input data identified the importance of features from August-September, which coincides with crop reproduction (Shook et al., 2021). The identification of features from explainable models provides biological understanding and supports results from other methods that identify important features. For example, the application of saliency maps as a post-hoc method to a convolutional neural network for soybean trait prediction identified an overlap with significant SNPs found from a GWAS conducted with the same data (Liu et al., 2019). Model explainability should be a consideration for genomic prediction problems and included as a determining factor for evaluating optimum model performance.

When predicting plant phenotypes from genotype information, the use of an interpretable model offers an opportunity to select high ranking markers as a feature selection strategy, and there is evidence that selecting a subset of important markers can improve the prediction for a given phenotype (Oakey et al., 2016). This is due to the large number of SNPs acting as background noise for prediction, resulting in diminishing returns on performance unless a major proportion of included SNPs are associated with the trait (Pérez-Enciso et al., 2015). A tool such as CGBayesNets can be used to firstly select a sample of features that are informative for phenotype prediction (McGeachie et al., 2014). Harvestman is another tool that selects a representative and non-redundant subset of features with a specific focus on minimising overfitting issues, which is common in high dimensionality prediction tasks (Frisby et al., 2021). The best subset and encoding of features can then be used to train new models. Ensemble methods can also be implemented, where interpretable ML methods can be used for feature selection, and the high ranking features can then be input into another model, such as a DL architecture, to improve predictions (Azodi et al., 2019). A benefit of feature selection is that input feature reduction can reduce the computing resources and time required to train models.

Model interpretation is complex, as definitions of interpretability are variable (Lipton, 2018), and evaluation of these interpretations is non-standardised, making evidence based comparisons between interpretable methods is difficult (Doshi-Velez and Kim, 2017). Rudin (2019) argues that instead of extracting meaning from the ‘black box’ model after training, ML models should be built with interpretability in mind (Rudin, 2019). Less complex ML algorithms, such as decision tree-based models, are inherently interpretable, with inbuilt functions to determine feature importance, however it can be hard to quantify importance between different models as the importance of these features often uses a metric that measures relative feature importance against other input features for a given model (Lundberg et al., 2018). For example, extreme gradient boosting’s (XGBoost) inbuilt functions offer multiple methods of calculating feature importance (weight, cover, gain), each offering different importance scores and ranking of input features, leading to variable interpretation of results depending on the method used. Model-agnostic local explanation methods, such as Shapley additive explanations (SHAP) (Lundberg and Lee, 2017) and Local interpretable model-agnostic explanations (LIME) (Ribeiro et al., 2016) have the potential to overcome this issue due to how the methods consistently and transparently quantify the input’s effect on prediction across most model types (Lundberg et al., 2018). However, this leads back to the original criticism of describing the extraction of meaning post-hoc from the black box as a practice with potential bias [57], the ability to purposely engineer explanations (Slack et al., 2020) and the likelihood of false conclusions being made by inexperienced users (Chromik et al., 2021).

Explainability for genotype to phenotype prediction is a relatively new area in genomic prediction studies, and the interest in explainability and interpretability suggests that new DL algorithms may have enhanced interpretability (Montesinos-López et al., 2021b). This presents a challenge to evaluate new algorithms, as conclusion extracted from interpretable models can be erroneous if the model’s predictive performance is poor (Murdoch et al., 2019). Additionally, interpretable ML extracts associations through the correlation between features and outcomes (Azodi et al., 2020), but are often interpreted with the goal of guiding hypotheses to suggest possible causal relationships from features (Lipton, 2018; Murdoch et al., 2019). Placed in a genotype to phenotype context, this would mean the identification of important genomic regions for phenotype outcome would remain noncausal and associative until further work was conducted to determine causality. One such example of why further work is required is the possibility of an untested factor not included in the genotype data, such as epigenetic or environmental features, that cause both the genomic region and the predicted phenotype to be associated with each other, without the genomic region being the causal feature.

## Discussion

ML models have the potential to predict complex phenotypic traits such as yield, biotic, and abiotic stress tolerance due to the way they capture the non-linear relationships between the genetic variation and environmental features extracted from the dataset. The growth of high throughput phenotyping and genotyping will continue to support crop breeding, however we need algorithms capable of handling data at this scale and complexity. Further research into the application of ML/DL models in crop breeding is required as the application of the model is highly dependent on representativeness of the training dataset, which may cause difficulties to apply the model in a real-world setting. Increased efforts to share datasets, or use data augmentation methods may help improve model generalisation. In addition, updating model weights as new datasets become available may also contribute to improve model prediction accuracy. Most genotype to phenotype studies currently lack interpretation of their predictions, which should be addressed to gain insight from the features. As researchers and breeders use advanced data analysis approaches, explainable ML models will help answer biological questions. Explainable MLwill be a key area for genotype to phenotype research, with an increasing focus on harnessing the potential of ML to generate accurate predictions combined with reliable interpretations.

## Abbreviations

DL, Deep Learning; GBLUP, Genomic Best Linear Unbiased Predictions; GS, Genomic Selection; GWAS, Genome-Wide Association Study; LASSO, Least Absolute Shrinkage and Selection Operator; LIME, Local Interpretable Model-agnostic Explanations; MIAPPE, Minimum Information About a Plant Phenotype Experiment; ML, Machine Learning; SHAP, SHapley Additive exPlanations; SNP, Single Nucleotide Polymorphism; RR, Ridge Regression; QTL, Quantitative Trait Loci.
